# PPIP5K1 Suppresses Etoposide-triggered Apoptosis

**DOI:** 10.5334/1750-2187-11-4

**Published:** 2016-11-23

**Authors:** Gayane Machkalyan, Terence E. Hèbert, Gregory J. Miller

**Affiliations:** Department of Pharmacology and Therapeutics, McGill University, Montréal, Québec, Canada; Department of Chemistry, The Catholic University of America, Washington, DC, USA

**Keywords:** inositol pyrophosphates, apoptosis, etoposide, inositol kinase, apoptosis array, p53

## Abstract

Inositol hexakisphosphate kinase 2 (IP6K2) potentiates pro-apoptotic signalling and increases the sensitivity of mammalian cells to cytotoxic agents. Diphosphoinositol pentakisphosphate kinase (PPIP5K) generates inositol pyrophosphates (InsPPs) that are structurally distinct from those produced by IP6K2 and their possible roles in affecting cell viability remain unclear. In the present study, we tested the impact of PPIP5K1 on cellular sensitivity to various genotoxic agents to determine if PPIP5K1 and IP6K2 contribute similarly to apoptosis. We observed that PPIP5K1 overexpression decreased sensitivity of cells toward several cytotoxic agents, including etoposide, cisplatin, and sulindac. We further tested the impact of PPIP5K1 overexpression on an array of apoptosis markers and observed that PPIP5K1 decreased p53 phosphorylation on key residues, including Ser-15, -46, and -392. Overexpression of a kinase-impaired PPIP5K1 mutant failed to protect cells from apoptosis, indicating this protection is a consequence PPIP5K1 catalytic activity, in contrast with the sensitivity conferred by IP6K2, which is dependent on both catalytic and non-catalytic functions. These observations reveal distinct roles for PPIP5K1 and IP6K2 and the InsPPs they produce in controlling cell death.

## Introduction

Inositol hexakisphosphate kinases (IP6Ks) comprise a highly conserved family that generates InsPPs in eukaryotic cells [1]. All IP6Ks add a phosphate to InP_6_ and thereby generate 5PP-InsP_5_ with a pyrophosphate moiety at position 5 of inositol ring [2,3]. The IP6K2 isoform of the IP6K family broadly mediates anti-proliferative and apoptotic effects of cytotoxic agents, including interferon-β (IFN-β), interferon-α2 (IFN-α2), and ionizing radiation, in numerous cultured cell lines [4,5,6]. These pro-apoptotic actions of IP6K2 occur through its interaction with p53 and subsequent transcription of pro-apoptotic proteins, including Noxa and Puma, while selectively suppressing transcription of p21 and other genes for pro-survival proteins [7].

PPIP5K generates 1PP-InsP_5_, with a pyrophosphate moiety on the 1-position. This molecule can be a substrate for IP6K, which thereby generates 1,5(PP)_2_-InsP_4_ [8]. PPIP5K regulates cell shape [8], growth, and phosphate homeostasis in yeast [9], is a positive regulator of interferon production [10] and affects cytoskeletal dynamics in *S. pombe* [11]. Little is known about the impact of PPIP5K1 on cell viability; however, there is evidence for a functional link between IP6K2 and PPIP5K activities, as both have been demonstrated to affect Akt activation, IP6K2 by affecting Akt localization itself, and PPIP5K1 through controlling localization of stress-activated MAP kinase-interacting protein1 (SIN1) [12]. Together, IP6K2 and PPIP5K1 coordinate formation of 1,5(PP)_2_-InsP_4_, so if this InsPP were a pro-apoptotic signal, then PPIP5K1 would act in concert with IP6K2 to promote cell death. We tested this hypothesis by investigating the impact of PPIP5K1 activity on cell viability and compared it to the known effects of IP6K2. We observed that PPIP5K1 overexpression confers cellular resistance to variety of cytotoxic stressors and protects cells from apoptosis, in contrast with IP6K2. PPIP5K1 activity diminished p53 activation in response to cellular stress and decreased both the expression of death receptors and signalling downstream from these receptors.

## Experimental Procedures

### Materials

All chemicals were reagent grade and purchased from Sigma Aldrich (Saint-Louis, Missouri, USA), unless stated otherwise.

### Molecular Biology

The human PPIP5K1 construct was generated by PCR amplification from PPIP5K1 cDNA acquired from the mammalian gene collection (ATCC-10436889) with primers 5’-GGGTGTACAGATGTGGTCATTGACGGCC-3’ and 5’-CCCGCGGCCG CCTAATTTATCTCCTCAGG-3’ (Integrated DNA Technologies, Coralville, IA, USA). The PCR product was digested with BsrGI and NotI (New England Laboratories, Ipswich, MA, USA) and cloned into the pIRESpuro-GLUE vector [13]. The PPIP5K1 kinase domain (residues 1–387) was PCR amplified using primers 5’-GGGGACAAG TTTGTACAAAAAAGCAGGCATGTGGTCATTGACGG-3’ and 5’-GGGGACCACTTTGTACAAGAAAGCTGGGTTTACTAATTTATCTCCTCAGGGACCTC-3’ and subsequently cloned into the Gateway pDONR221 vector and the pDEST-58 expression vector (Life Technologies, Carlsbad, CA, USA). A synthetic, codon-optimized gene for human IP6K2 was assembled and subsequently amplified by PCR using primers 5’-GGGTGTACAGATGAGCCCAGCCTTCAGGGCC-3’ and 5’-CCCGCGGCCGCTC ACTCCCCACTCTCCTCACTTA-3’ [14]. This PCR product was digested with BsrGI and NotI and cloned into the pIRESpuro-GLUE vector [13]. For bacterial expression, IP6K2 was amplified by PCR with 5’- GGGAATTCGATGAGCCCAGCCTTCAGG-3’ and 5’- CCCTCGAGGTCACTCCCC ACTCTCCTC-3’. The amplified product was digested with EcoRI and XhoI (New England BioLabs) and cloned into pET-SUMO (a generous gift from Dr. C. Lima, Memorial Sloan Kettering Cancer Center [15]). The Quick Change II kit (Agilent Technologies, Santa Clara, CA, USA) was used to introduce mutations in PPIP5K1 and IP6K2. The PPIP5K1^K259A^ mutant was generated using primers 5’- GCCCCACTGTATACACCGCGACATCTGTGCCATCTG-3’ and 5’-CAGATGG CACAGATGTCGCGGTGTATACAGTGGGGC-3’. All constructs were verified by bidirectional sequencing (IRCM Molecular Biology and Functional Genomics Facility, Montreal, Quebec, Canada).

### Cell lines

Human embryonic kidney 293 (HEK 293F) cells were maintained in Dulbecco’s modified Eagle’s medium (DMEM) (Wisent, St-Bruno, Quebec, Canada) supplemented with 5% FBS (Wisent) at 37 °C with 5% CO_2_. Cells were transfected with Lipofectamine 2000 as per manufacturer’s instructions (Life Technologies). Stable expression of PPIP5K1^WT^, PPIP5K1^K259A^, and IP6K2 was induced by selection with 1 μM puromyocin (InVivoGen, San Diego, California) 48 hours post transfection. After testing for expression of each construct, polyclonal cell lines were expanded and maintained in the presence of puromycin.

### Cell lysis

HEK 293F cells stably expressing PPIP5K1 (wild-type or K259A) or IP6K2 were grown on 6 well plates. When cells reached 80% confluence, the plates were placed on ice and cells were washed twice with phosphate-buffered saline (PBS) (137 mM NaCl, 2.7 mM KCl, 10 mM Na_2_HPO_4_, 2 mM KH_2_PO_4_), and the cells were resuspended in 300 µl radioimmunoprecipitation assay (RIPA) buffer (50 mM Tris-HCl, pH 7.4, 150 mM NaCl, 1% NP-40, 1% sodium deoxycholate, 0.1% SDS, 1 mM EDTA, 1 mM EGTA, 1 mM DTT, 2 mM NaF, 2 mM NaVO_3_, 1 mM PMSF), as well as a commercially available protein inhibitor cocktail (Roche Diagnostics, Indianopolis, IN, USA). Cell lysates were incubated for 1 hour with gentle rotation at 4 °C, then were sonicated and cleared by centrifugation at 3 000 × *g* for 10 minutes. Protein concentrations were measured using the Bio-Rad protein assay (Mississauga, Ontario, Canada) using BSA as a standard and 25 μg of protein were used for western blot analysis.

### Flow cytometry

HEK 293F expressing PPIP5K1^WT^ or IP6K2, or mock-transfected control cells were plated (1 × 10^5^ cells/well) on 6 well plates. Cells were treated for 16 hours with either 25 μM etoposide or DMSO. After treatment, cells were washed twice with Dulbecco’s phosphate-buffered saline (DPBS) (0.9 mM CaCl_2_, 0.5 mM MgCl_2_, 2.7 mM KCl, 1.47 mM KH_2_PO_4_, 138 mM NaCl, 8 mM Na_2_HPO_4_) and labeled using the Annexin V-FITC Apoptosis Detection kit (Sigma Aldrich, Saint-Louis, Missouri, USA) as suggested by the manufacturer. A Becton Dickinson FACSCan automated flow cytometer (McGill Flow Cytometry Core Facility, Montreal, Quebec, Canada) was used to sort cells and Becton Dickinson Cell Quest Pro software was used to collect and analyze the data. Statistical analysis was performed using data from independent experiments conducted in triplicate.

### High content viability assay

Cell counts were performed using an automated TC20 cell counter (BioRad) and 1.5 × 10^4^ cells were plated in quadruplicate in wells of black Costar clear-bottom 96 well plates (Corning Incorporated, Corning, NY, USA) coated with 0.1 mg/mL poly-D-lysine (Sigma Aldrich), and allowed to attach overnight under normal growth conditions. Etoposide, cisplatin, camphothecin (Sigma Aldrich), and 5-fluorouracil (EMD Millipore Corporation, Billerica, MA) were diluted with DMSO and added to DMEM supplemented with 5% FBS at the indicated concentrations. After treatment, cells were double stained with 5 μg/ml Hoesch33342 (Sigma Aldrich) and 10 μg/ml propidium iodide (PI) for 10 minutes. Using an Operetta High Content Microscopy System (Perkin Elmer, Waltham, MA, USA), nine fields per well were imaged using confocal 10X objective with Hoeschst3342 filter (excitation filter 360–400 nm; emission filter: 410–480 nm) and PI filter (excitation filter: 560–580 nm; emission filter: 590–640 nm). Columbus Image analysis software (Perkin Elmer, Waltham, MA, USA) was first trained to count cells in each well by identifying nuclei stained with Hoesch33342, then nuclei stained with PI were counted and cell viability was measured as the percentage of PI positive cells of the total number of cells. Statistical analysis was performed using data from three or more independent experiments.

### Protein expression and purification

Wild type and K259A mutant kinase domains of PPIP5K1 (1–387) were expressed in *E.coli* BL-21 Rosetta cells (Novagen, Madison, WI, USA). Cells were grown to an OD_600_ = 0.8 before induction using 0.5 mM IPTG for 16 hours at 16 °C. Cells were subsequently pelleted at 300 × g, resuspended in 50 mM Tris-HCl, pH 7.4, 300 mM NaCl, 30% glycerol, 1.7 mM β-mercaptoethanol, and then lysed by sonication. The soluble fraction was separated from the insoluble by centrifugation at 10 000 × *g*. The supernatant was then diluted two-fold with 50 mM Tris-HCl, pH 7.4, 500 mM NaCl, and 30 mM imidazole. Proteins were purified using Co^2+^-NTA beads (Thermo Fisher Scientific, Rockford, IL, USA) using 500 μl of dry beads per 250 ml of bacterial culture. After loading the soluble fraction onto the beads, they were washed with 50 mM Tris-HCl, pH 7.4, 800 mM NaCl, 1% Triton X-100, and 1.7 mM β-mercaptoethanol. Proteins were eluted using 50 mM Tris-HCl, pH 7.4, 150 mM NaCl, and 250 mM imidazole. After elution, 1 mM DTT was added to the eluates. Proteins were concentrated using Amicon Ultra centrifugal filter devices (EMD Millipore Corporation) then the buffer was exchanged to 50 mM HEPES pH 7.4, 150 mM NaCl, 0.5 mM EDTA, and 1 mM DTT. Protein concentrations were measured by Bradford protein assay (Bio-Rad) using BSA as standard. Protein was stored at 4 °C and used within 48 hours.

### Kinase assay

PPIP5K1 and mutant kinase activities were assessed using Kinase Glow Max Luminescent Assay (Promega Corporation, Madison, WI, USA). Kinase reactions were modified from a protocol published previously [16] and were performed in 25 μl in black 96 well plates (Corning Incorporated) at 25 °C. The reaction mixtures contained 50 mM HEPES pH 7.5, 150 mM KCl, 1mM DTT, 0.2 mM EDTA, 2 mM MgCl_2_, IP_6_, and purified enzyme (0.5–2 μg). Reactions were stopped by the addition of 25 μl Kinase-Glo reagent and luminescence was measured after 10 minutes using a Berthold Orion II Microplate Luminometer. Negative control reactions included enzyme, but lacked IP_6_ substrate. Activity of the enzyme was assessed by measuring relative ATP consumption upon addition of enzyme in the presence and absence of IP_6_ [16].

### Apoptosis array

Cells expressing PPIP5K and mock-transfected HEK 293F control cells were grown on 10 cm^2^ plates to 70% confluence. Cells were treated either with DMSO or 25 μM etoposide for 16 hours. Cells were then harvested and the expression of apoptosis-related proteins was measured using the Human Apoptosis Antibody Array kit (Thermo Fisher Scientific) according to manufacturer instructions. The total protein concentration in each sample was measured by Bradford protein assay (Bio-Rad) using BSA as standard. Equal amounts of total protein from each lysate was incubated with each array and incubated at 4 °C overnight with gentle rocking. Arrays were incubated with biotinylated secondary antibodies for 1 hour, followed by incubation of streptavidin-HRP for 30 minutes. Chemiluminescence was used to develop signals that were visualized on autoradiography film (HyBlot CL, Metuchen, NJ, USA). Western blots were analyzed using ImageJ image analysis software (NIH). Statistical analysis was performed on data from three independent experiments. Mixed repeated measures two-way ANOVA was done on each apoptosis marker to compare control and PPIP5K1 stable cell lines treated with either vehicle or etoposide.

### Reverse transcription and qRT-PCR

Mock-transfected HEK 293F control cells, PPIP5K1- and IP6K2-expressing stable cell lines were plated at 1 × 10^5^ cells/well on 6 well plates and allowed to grow to 70% confluence and were then treated either with DMSO or 25 μM etoposide for 16 hours. Total RNA was isolated from treated cells with TRIzol reagent using modified RNA isolation protocol Ambion (Life Technologies). Reverse transcription and qRT-PCR protocol used were described previously [17]. Death receptor 4 (DR4) and death receptor 5 (DR5) mRNA expression was assayed in duplicate and compared to β-actin expression. Primers used for real time qRT-PCR were designed using NCBI tool Primer Blast, which were validated for selectivity for a single product by assessing dissociation curves. Primers used for qRT-PCR of DR4 mRNA were 5’-ACGAGATTCTGAGCAACGCA-3’ and 5’-CAGCACCATTTG CTGGAAC-3’ and for DR5 mRNA were 5’- CCCTGT TCTCTCTCAGGCATC-3’ and 5’-CAGGTCGTT GTGAGCTTCTGT-3’.

### Data analysis

Data are presented as mean ± S.E.M. Intensity of bands from western blot analysis were measured by densitometry using ImageJ [18]. Statistical analyses were performed with GraphPad Prism software (version 5.0 for Mac OS X, San Diego, CA, USA) using paired t-test, one-way analysis of variance, or two-way analysis of variance with Bonferroni post-hoc tests. Statistical significance was defined as p values < 0.05.

## Results

### PPIP5K1 protects cells from apoptosis

**Figure 1:**
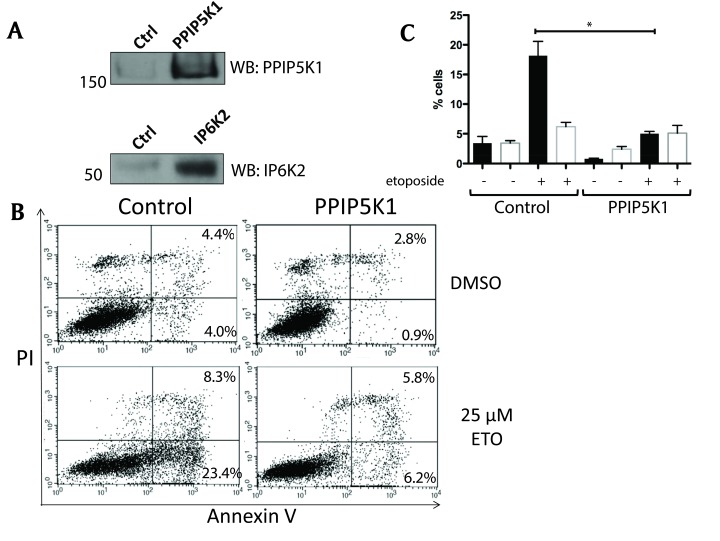
Flow cytometry analysis of apoptosis PPIPK1-expressing cells. **A.** Untransfected HEK 293F control and stable cell lines expressing PPIP5K1 and IP6K2 were subjected to immunoblot analysis using anti-PPIP5K1 and anti-IP6K2 antibodies. **B.** Representative data from flow cytometry from three independent experiments. HEK293F untransfected (control) and stable cell lines were incubated either with DMSO or 25 µM etoposide for 16 hours. All attached and detached cells were collected and double stained with Annexin V and propidium iodide (PI) using APOAF kit from Sigma following manufacture’s instructions. Percentage of healthy (unstained), early apoptotic cell (Annexin V only), late apoptotic (Annexin V and PI) and necrotic (PI only) cells were determined. **C.** Percentages of cells in early apoptosis (black bars) and late apoptosis, as determined from three independent flow cytometry experiments.

IP6K2 has been reported to promote apoptosis, and yet also drives tumor growth and metastasis [4]. To investigate the impact of PPIP5K1 on cell viability and sensitivity to cytotoxic agents, we expressed PPIP5K1 in HEK 293F cells and challenged these cells with cytotoxic agents. After confirming the expression of PPIP5K1 in stably expressing cell lines (Figure 1A), we compared the responses of these cell lines when challenged by the topoisomerase II inhibitor and DNA damaging agent etoposide (25 μM) [19]. We measured early and late stages of apoptosis by Annexin V and propidium iodide (PI) staining, using flow cytometry. Overexpression of PPIP5K1 significantly reduced the sensitivity of cells to etoposide compared to control cells with a marked decrease in the number of cells in early apoptosis after etoposide treatment (Figure 1B, C). Effects of PPIP5K1 were limited to etoposide-challenged cells, as it did not alter cell viability in the absence of etoposide (Figure 1B and 1C).

**Figure 2:**
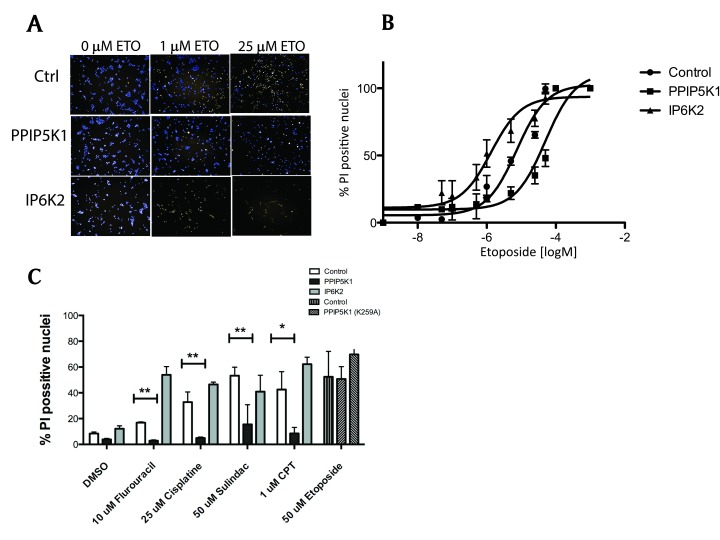
Analysis of apoptosis in PPIP5K-1 and IP6K2-expressing cells. **A.** HEK 293F control and stable cell lines were grown in 96 well plate, treated with 0, 1 µM and 25 µM etoposide for 16h. Cells were double stained with Hoeschst33342 (5 mg/ml) and propidium iodide (PI) (10 mg/ml) for 10 min and imaged in Operetta (Perkin Elmer) high content microscope. **B.** Dose-response curve of three cell lines treated with varying concentrations of etoposide for 16 h; Percentage of PI positive nuclei is used to asses cell viability; the graph is a curve of a single experiment of three independent experiments. **C.** Cell viability of HEK 293F control, and PPIP5K1, IP6K2, or PPIP5K1^K295A^ stable cell lines treated with indicated concentrations of cisplatin, sulindac, camptothecin (CPT), 5-Fluorouracil, or etoposide for 3h and stained as described above. The graph is a single experiment, representative of three independent experiments. Two-way ANOVA test with Bonferroni post-test was used to compare p values between groups (**p < 0.01; ***p < 0.001).

We extended our viability studies to compare the impact of IP6K2 and PPIP5K1 expression on apoptosis using high-content microscopy to measure cell death as percentage of PI-positive nuclei using Hoeschst 33342 as a counterstain. Consistent with observations made using flow cytometry, overexpression of PPIP5K1 decreased the percentage of PI-positive cells in etoposide-treated samples (Figure 2A and 2B). PPIP5K1 decreased sensitivity of HEK 293F cells to etoposide treatment by 2.7-fold, while expression of IP6K2 increased sensitivity of cells by more than 2-fold (Figure 2B).

As IP6K2 is broadly protective against a range of apoptosis-inducing agents, we tested several cytotoxic agents to determine if PPIP5K1 has similar broad range of effects. 5-Fluorouracil cisplatin, sulindac, and camptothecin (CPT) all were markedly less effective at inducing cell death in PPIP5K1-expressing cells than control cells (Figure 2C). Over expression of IP6K2 made these cells highly sensitive to all of these agents in agreement with previous reports.

Next, we evaluated whether PPIP5K1 catalytic activity was required for these differences. We mutated and tested PPIPK1 to have a Lys259Ala mutation, because of the critical catalytic role this residue appears to play based on the crystal structure of the PPIP5K2 kinase domain [20]. We measured viability of cells expressing PPIP5K1^K259A^ and observed no significant difference between cells expressing the kinase-dead PPIP5K1 and control HEK 293 cells, which indicated less resistance to apoptotic stressors compared to PPIP5K1^WT^ when cells were treated with etoposide (Figure 2C). These observations indicate the catalytic activity of PPIP5K1 is required for its anti-apoptotic effects.

### Impact of PPIP5K1 and IP6K2 on apoptosis signalling

**Figure 3:**
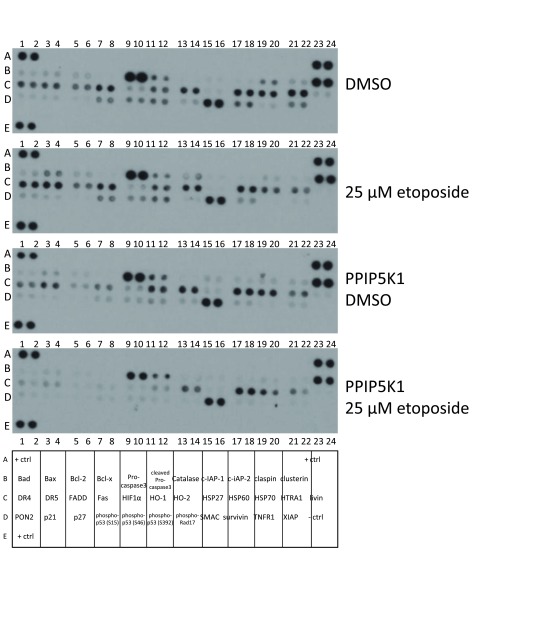
Overexpression of PPIP5K1 impacts apoptosis signaling. A. Apoptosis array of control and PPIP5K1-overexpressing cells. Results are representative of three independent experiments. B. Map of individual proteins in the apoptosis array.

**Figure 4:**
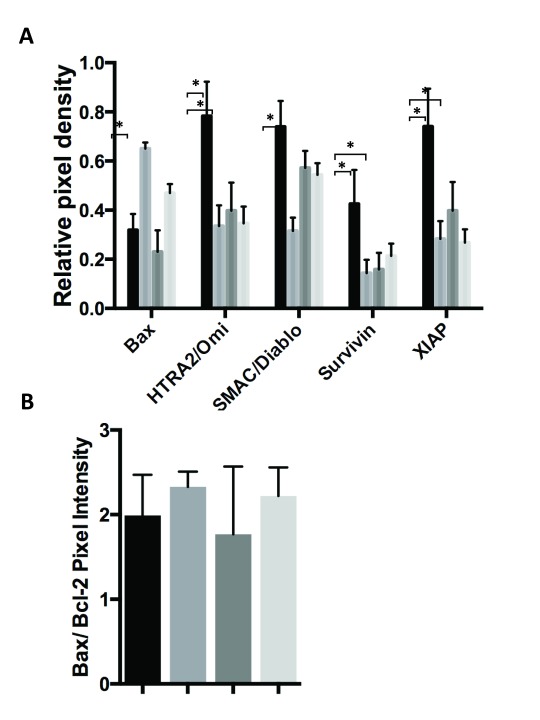
Overexpression of PPIP5K alters levels of proteins that control cell death. A. Apoptosis arrays of control and PPIP5K1-overexpressing cells were analyzed in triplicate using ImageJ software to identify those markers with altered expression in PPIP5K1-overexpressing cells and when treated with etoposide. The Fold change is the ratio of the average pixel density from etoposide treated cells to the average pixel density of DMSO treated cells for each marker measured in triplicate from three independent experiments. B. Apoptosis arrays of control and PPIP5K1-overexpressing cells were analyzed to determine the ratio of Bax and Bcl-2 pixel intensities. The ratio and the SD are shown for control cells treated with DMSO, control cells treated with etoposide, PPIP5K1-over expressing cells treated with DMSO, and PPIP5K1-overexpressing cells treated with etoposide.

**Figure 5:**
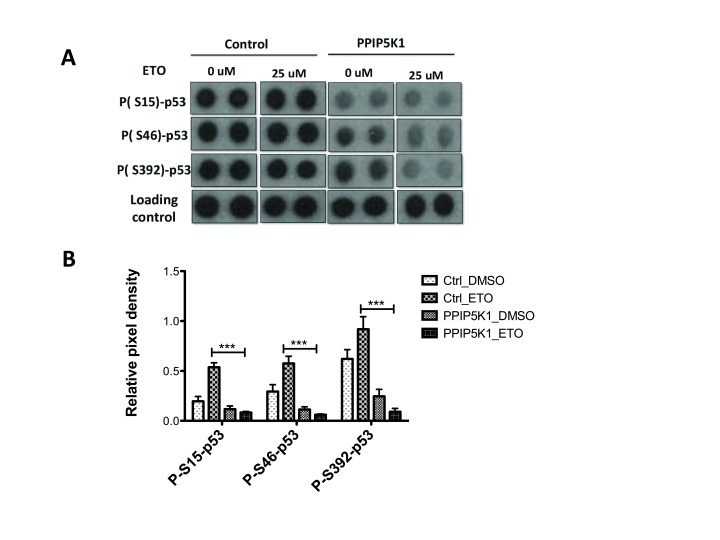
PPIP5K1 impacts p53 pathway phosphorylation. A. Protein apoptosis array of control and PPIP5K1 stable cell lines treated either with DMSO or 25mM etoposide for 16 h. An ECL developed apoptosis array film images of p53 phosphorylated markers along with loading controls of control and PPIP5K1 stable cell lines treated with either vehicle or etoposide. B. Bar graph showing relative pixel density of p53-phospho markers in the control and PPIP5K1 cells treated either with DMSO or etoposide. ImageJ software was used to measure pixel densities of each marker and relative pixel density was calculated as ratio of a signal to a loading control of each array. Relative pixel densities from three independent experiments were combined to make the graphs and two-way ANOVA statistical test was used to compare control and PPIP5K1 cells. Bonferroni post hoc test was used to compare groups and statistical significant difference is shown- Control_ETO vs PPIP5K1_ETO ( p < 0.001) for all 3 phospho-p53 markers.

**Figure 6:**
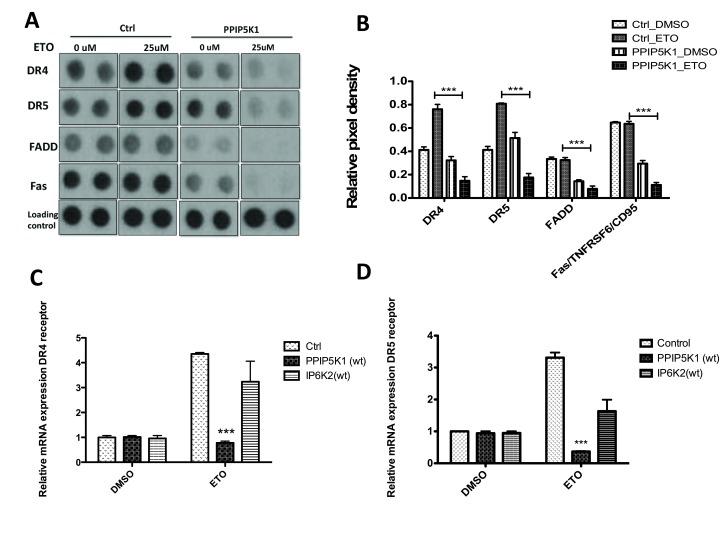
PPIP5K1 and IP6K2 impact on Death receptor expression. **A.** Protein apoptosis array of control and PPIP5K1 stable cell lines treated either with DMSO or 25 µM etoposide for 16 h. A ECL-developed apoptosis array film images of DR4, DR5, FADD and Fas proteins along with loading controls of control and PPIP5K1 stable cell lines treated with either vehicle or etopoiside. **B.** Bar graph showing relative pixel density of DR4, DR5, FADD, and Fas proteins in the control and PPIP5K1 cells treated either with DMSO or etoposide. ImageJ software was used to measure pixel densities of each marker and relative pixel density was calculated as ratio of a signal to a loading control of each array. Relative pixel densities from three independent experiments were combined to make the graphs and two-way ANOVA statistical test was used to compare control and PPIP5K1 cells. Bonferroni post hoc test was used to compare groups and statistical significant difference for control_ETO vs PPIP5K1_ETO ( p<0.001) for DR4, DR5, FADD and Fas proteins is shown. **C.** and **D.** Relative mRNA expression levels of DR4 (panel C) and DR5 (panel D) receptors in control, PPIP5K1 and IP6K2 cells treated either with vehicle or 25 mM etoposide for 16 h.

To further investigate the impact of PPIP5K1 on apoptosis, we compared 34 different apoptosis markers upon etoposide treatment in control cells and cells expressing PPIP5K1 (Figure 3). First, we compared cells expressing PPIP5K1 with control cells when both were similarly-treated with DMSO and discovered that PPIP5K1-expressing cells showed decreased amounts of apoptosis markers that contribute to apoptosis, including Bax, HTRA2 (Figure 4A), p53 phosphorylated at at Ser-15, -46, and -392 (Figure 5), DR4, FADD, and Fas (Figure 6). These data indicate PPIP5K1 expression alters apoptotic machinery of non-stimulated cells. As we observe changes in apoptosis and apoptosis markers, we compared the ratios of Bax and Bcl-2 in each apoptosis array, but we did not detect significant changes to this ratio in any of the experimental conditions (Figure 4B)

Next, we compared responses to etoposide of PPIP5K1 overexpressing cells and control cells. We observed decreased levels of pro-apoptotic markers, including p53 phosphorylated at Ser-15, -46, and -392 (Figure 5), DR4, DR5, and Fas (Figure 6) following etoposide treatment of PPIP5K-expressing cells, but which were up-regulated in control cells following similar treatment.

Using the apoptosis marker array, we observed that PPIP5K1 overexpression resulted in decreased basal levels of multiple components of the death receptor pathway (i.e., TNF, DR4, DR5, FADD and Fas) compared to control cells, which were further decreased upon etoposide treatments (Figures 3 and 6A, B). In control cells, etoposide treatment increased levels of death receptor expression, which is consistent with previous reports [21]. However, in PPIP5K1 cells, DR4 and DR5 levels were decreased substantially and decreased further with etoposide treatments (Figure 6B). We also observed that mRNA levels of DR4 and DR5 were were unchanged with PPIP5K1 expression, but decreased in PPIP5K1-expressing cells when they were treated with etoposide, which was consistent with observed protein levels using the apoptosis antibody array (Figure 6C, D). In contrast, etoposide treatments of IP6K2-overexpressing and control cells increased DR4 and DR5 mRNA levels (Figure 6C, D). Taken together, our results show that these two enzymes differ greatly in how they modulate responses to apoptotic stressors.

## Discussion

In this study, we demonstrate a previously unrecognized role for PPIP5K1 in regulating cell death in response to genotoxic stress. We observed that overexpression of PPIP5K1 in HEK 293 cells protected them from apoptosis triggered by etoposide and other agents, in contrast to overexpression of another InsPP -generating enzyme, IP6K2, which sensitizes cells to cytotoxic agents [5,6,7,22,2322, 23]. After measuring the impact of PPIP5K1 on an array of apoptosis-related proteins, we also observed that PPIP5K1 alters phosphorylation of p53 at three key residues, which suggests a mechanism through which PPIP5K1 acts.

We observed the protection provided by PPIP5K1 to be dependent on the production of InsPP, as the anti-apoptotic activity provided was lost in cells expressing a kinase-impaired mutant. Previous reports have indicated that total cellular levels of InsPP are not significantly affected by over expression of PPIP5K1. We therefore cannot unambiguously conclude that it is the production of 1-PP-IP_7_ that is the direct agent responsible for changes in apoptosis that we observe. We have recently reported that PPIP5K1 interacts with the exocyst complex and others have observed its localization at the plasma membrane in response to cell stimuli [24]. These observations suggest that PPIP5K1 acts at discrete sites and localized increases of InsPP may affect cellular processes without markedly affecting total cellular InsPP levels. The three isoforms of IP6K all perform the same catalytic reaction and have different cellular localization patterns, with only IP6K2 sensitizing cells to cytotoxic stress, which suggests it is the location of InsPP generation that is key for this effect. PPIP5K has two isoforms that also localize differently, so it is possible, that PPIP5K1 and PPIP5K2 will affect apoptosis signalling differently. Alternately, the active signal may not be the 1PP-InsP_5_ directly produced by PPIP5K, but may be a product of its further phosphorylation by IP6K2 to create a new apoptosis-affecting signals [8,25] or PPIP5K1 may inactivate the apoptosis-promoting signal generated by IP6K2 by phosphorylating its products. It remains to be determined whether IP6K2 and PPIP5K can both generate distinct active signals or sequentially induce and then inactivate a single signal.

Phosphorylation of p53 at three key residues, Ser-15, -46, and -392 lead to increases in cellular p53 levels by disrupting p53 interaction with MDM2, the p53 ubiquitin ligase. We observed decreased levels of p53 phosphorylation at all of these sites in PPIP5K1 over-expressing cells. Challenging these cells with etoposide further decreased p53 phosphorylation, indicating phosphorylation is not simply decreased generally, but specifically when cells are exposed to genotoxic stress. It is noteworthy that IP6K2 induces apoptosis in response to etoposide and other agents in a manner leading to increased p53 phosphorylation. IP6K2 binds to CK2 and its product, IP_7_, activates it, which stabilizes Tti1/Tel2, a chaperone complex, and ATM, which phosphorylates p53 [22]. Our observation that PPIP5K1 leads to the opposite response, with decreased p53 phosphorylation and decreased apoptosis, suggests PPIP5K1 may either be generating a novel-acting signal, or may be inactivating the apoptosis-controlling signal generated by IP6K2.

We have recently observed that PPIP5K1 interacts directly with components of the exocyst complex and is involved in the regulation of cellular motility [24]. The assembly of the exocyst complex is regulated by Ras-like GTPases, including RalA, which we also identified as a PPIP5K-interacting protein [26]. Ral GTPases protect cells from genotoxic stresses by a mechanism apparently independent of exocyst complex actions [27]. Our observations that PPIP5K1 binds to the exocyst complex, RalA, and has anti-apoptotic properties suggest a possible link between PPIP5K and Ral GTPase-mediated tumor development. Further investigations are needed to explore the possible roles of PPIP5K1 actions in Ras-mediated oncogenesis.

## Conclusions

In the present study we report the impact of PPIP5K1 enzymatic activity on cellular viability. PPIP5K1 overexpression increases the tolerance of mammalian cells to various genotoxic stressors. Our observations that elevated PPIP5K activity decreases signalling through the p53 pathway which suggests that PPIP5K inhibition may be a path to sensitize cells to anti-cancer and apoptosis-inducing therapeutics.
